# Curation of the Mammalian Palmitoylome Indicates a Pivotal Role for Palmitoylation in Diseases and Disorders of the Nervous System and Cancers

**DOI:** 10.1371/journal.pcbi.1004405

**Published:** 2015-08-14

**Authors:** Shaun S. Sanders, Dale D. O. Martin, Stefanie L. Butland, Mathieu Lavallée-Adam, Diego Calzolari, Chris Kay, John R. Yates, Michael R. Hayden

**Affiliations:** 1 Centre for Molecular Medicine and Therapeutics, Department of Medical Genetics, Child & Family Research Institute, University of British Columbia, Vancouver, British Columbia, Canada; 2 Department of Chemical Physiology, The Scripps Research Institute, La Jolla, California, United States of America; Tel Aviv University, ISRAEL

## Abstract

Palmitoylation involves the reversible posttranslational addition of palmitate to cysteines and promotes membrane binding and subcellular localization. Recent advancements in the detection and identification of palmitoylated proteins have led to multiple palmitoylation proteomics studies but these datasets are contained within large supplemental tables, making downstream analysis and data mining time-consuming and difficult. Consequently, we curated the data from 15 palmitoylation proteomics studies into one compendium containing 1,838 genes encoding palmitoylated proteins; representing approximately 10% of the genome. Enrichment analysis revealed highly significant enrichments for Gene Ontology biological processes, pathway maps, and process networks related to the nervous system. Strikingly, 41% of synaptic genes encode a palmitoylated protein in the compendium. The top disease associations included cancers and diseases and disorders of the nervous system, with Schizophrenia, HD, and pancreatic ductal carcinoma among the top five, suggesting that aberrant palmitoylation may play a pivotal role in the balance of cell death and survival. This compendium provides a much-needed resource for cell biologists and the palmitoylation field, providing new perspectives for cancer and neurodegeneration.

## Author Summary

Protein localization is essential for mediating protein function within the cellular context. Mislocalization of proteins can offset cellular balance, influencing whether a cell lives or dies. Many proteins are directed to cellular membranes through the addition of fats, or lipidation. In particular, palmitoylation involves the reversible addition of the fatty acid palmitate to cysteines. Its reversibility makes it a unique form of lipidation allowing its dynamic regulation. Recent advancements in fast, sensitive, non-radioactive methods to detect palmitoylation have led to an explosion in the identification of palmitoylated proteins through proteomics studies. However, the data is hidden in large supplemental tables in various formats. Thus, we curated a list of palmitoylated proteins revealing that approximately 10 percent of the human genome encodes for a proteoform that is palmitoylated. Computational analysis confirmed that palmitoylation is involved in protein localization and indicated a new role in metabolism. Importantly, we found that palmitoylation was enriched at neuronal synapses and in disorders of the nervous system, including Schizophrenia and Huntington disease. Interestingly, palmitoylation was equally enriched in cancers. Consequently, we suggest that palmitoylation plays a critical role in cell fate and our compendium provides a plethora of targets for neurodegeneration and cancer.

## Introduction

**Fig 1:**
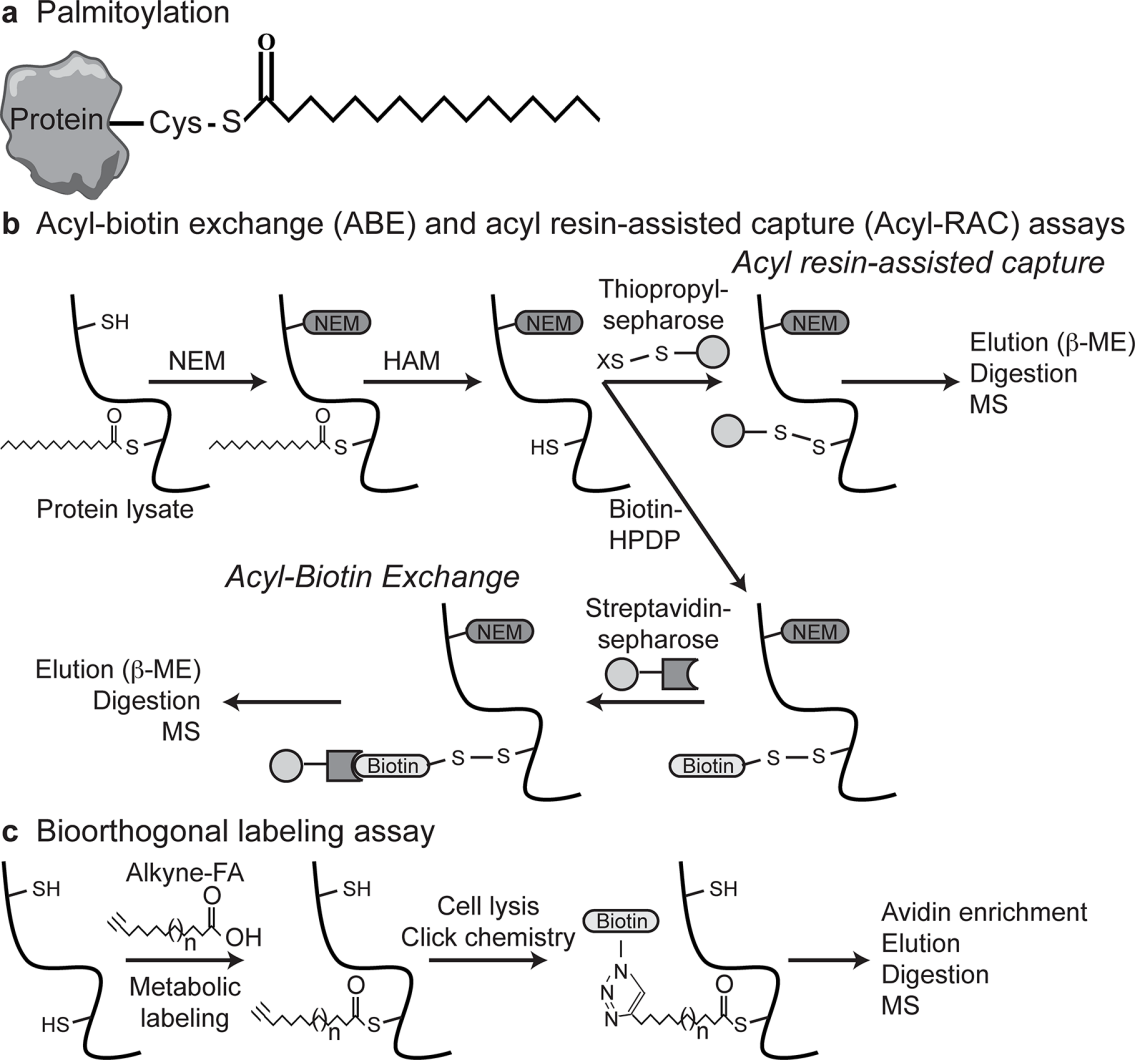
Palmitoylation and detection methods. (**a**) Palmitoylation involves the reversible addition of long-chain fatty acids (FA) to cysteine residues via thioester bonds. (**b**) The ABE and Acyl-RAC assays use N-ethylmaleimide (NEM) to block free cysteines and hydroxylamine (HAM) to remove palmitate. The Acyl-RAC assay uses thiopropyl-sepharose beads that covalently react with the free cysteines, allowing enrichment and elution, using β-mercaptoethanol (β-ME), of palmitoyl-proteins and detection by MS. Following HAM treatment in the ABE assay free cysteines are labeled using Biotin-HPDP, allowing streptavidin-sepharose enrichment for MS. (**c**) The bioorthogonal-labeling assay uses alkyne-FA analogues followed by click chemistry to covalently link alkynyl-palmitate with biotin, allowing enrichment of palmitoyl-proteins for MS.

S-Acylation (commonly referred to as palmitoylation) involves the reversible post-translational addition of long-chain fatty acids, typically palmitate, to cysteine residues of both peripheral and integral membrane proteins by palmitoyl acyltransferases (PATs; Fig 1A) [1,2]. Palmitoylation increases the hydrophobicity of a protein and thereby promotes membrane binding, regulates subcellular localization and protein stability, induces tilting of transmembrane domains, and modulates protein-protein interactions [3]. While the fatty acid moiety is typically associated with membrane association, palmitoylation has also been shown to regulate the active cysteines of enzymes [4]. In mammals, palmitoylation is mediated by 23 DHHC-domain containing PATs [5–8]. While palmitoylation can be highly dynamic in some proteins due to its reversibility, many proteins have been found to be stably palmitoylated and retain their palmitate. Dynamic depalmitoylation is mediated by acyl protein thioesterases in the cytosol [9,10]. Therefore, the reversible nature of palmitoylation, which is analogous to that of phosphorylation, can add another layer of regulation to promote “on/off” states of membrane association or activity.

Alterations in PAT activity or palmitoylation of specific proteins have been implicated in a number of diseases, including cancer [11–15], diabetes [16,17], Schizophrenia [18–20], X-linked mental retardation [21,22], and neurodegeneration, including Alzheimer disease [23–25], Huntington disease [8,26], and Amyotrophic Lateral Sclerosis (AD, HD, and ALS, respectively) [27,28]. Palmitoylated proteins previously implicated in neurodegeneration include APP [25], BACE1, APH1, nicastrin, HTT [29], and SOD1 [27,28].

Recently, a number of studies have focused on determining the “palmitoylome” in diverse cell types to determine the role of palmitoylation in various processes including cancer, immunity, and synaptic function. Sixteen mammalian palmitoylation proteomics studies have been described to date in rat, mouse, and human cells, including endothelial, immune, and neuronal cells, as well as mouse brain tissue (Table 1). Three assays were used to detect palmitoylation in these studies: acyl-biotin exchange (ABE) [30,31], acyl resin-assisted capture (Acyl-RAC) [32], and bioorthogonal labeling assays [33,34] (Fig 1B and 1C). The two former assays exploit the reversibility of palmitoylation and the reactivity of cysteines to replace the palmitate moiety with biotin for affinity purification and mass spectrometry (MS) analysis. The latter assay uses long-chain fatty acid analogs, similar to radioactive labeling with iodinated or tritiated palmitate that, can be chemically ligated to biotin. The majority of the data generated by these studies have been overlooked since they are contained within large supplemental tables where proteins are described with different types of identifiers, making downstream analyses and data mining of the combination of datasets time consuming and inaccessible to many researchers without bioinformatics expertise. Searching the supplemental data of these studies to determine if a protein of interest may be palmitoylated is a tedious and time consuming task. Therefore, we curated the palmitoylated proteins identified in these proteomics studies into a consolidated non-redundant searchable list. This compendium provides a valuable resource for those working in the field of palmitoylation and for the wider research community that may be interested in the post-translational regulation of a given protein of interest. In addition, enrichment analysis of this compendium provides the first unbiased approach to understanding the role of palmitoylation in cell biology and disease.

**Table 1:** Published mammalian palmitoylomes.

Organism	Cell or Tissue Type	Cell Fraction	Detection Method	Reference	Data Source
Human					
	HUVEC endothelial cells	total cell lysate	ABE-NEM	Wei *et al*. *ATVB*. 2014.	Union of Online Tables II and IV
	B lymphocytes	membrane lysate	ABE-NEM	Ivaldi *et al*. *PLoS ONE*. 2012.	Supp. S3 Table
	endothelial cell line EA.hy926	total cell lysate	ABE-MMTS	Marin *et al*. *Circ Res*. 2012.	Supp. S1 Table
	resting platelets	membrane lysate	ABE-NEM	Dowal *et al*. *Blood*. 2011.	Supp. S3 Table, p<0.05
	HEK293 cells	membrane lysate	acyl-RAC-MMTS	Forrester *et al*. *JLR*. 2011.	Supp. S1 Table
	Jurkat T cells	total cell lysate	Bioorthogonal labeling (myristic: az-12 or 13-TDYA; palmitic: 15-HDYA; stearic: az-15 or 17-ODYA)	Wilson *et al*. *MCP*. 2011.	Supp. S1A Table, Supp. S2A Table (i.e. take both high and medium confidence hits)
	prostate cancer cell line DU145	lipid raft and non-lipid raft membrane fractions	ABE-NEM	Yang *et al*. *MCP*. 2010.	Supp. S2 Table high confidence, Supp. S3 Table med confidence
	Jurkat T cells	membrane lysate	Bioorthogonal labeling (stearic: 17-ODYA) + HAM	Martin *et al*. *Nat Methods*. 2009.	Supp. S1 Table High confidence, Supp. S2 Table Medium confidence
	HeLa cells	membrane lysate	acyl-RAC-MMTS	Zhang *et al*. *MCP*. 2008.	Supp. S1 Table
Mouse					
	brain	total cell lysate	ABE-NEM	Wan *et al*. *Chem & Bio*l. 2013.	S1 Table, S2 Table
	T-cell hybridoma cells	membrane lysate	Bioorthogonal labeling (stearic: 17-ODYA) + HAM	Martin *et al*. *Nat Methods*. 2012.	Supp. S2 Table
	neuronal stem cells	membrane lysate	Bioorthogonal labeling (stearic: 17-ODYA) + HAM	Li *et al*. *JBC*. 2012.	S1 Dataset, Supp. S5 Table, labelled "BOTH"
	macrophage cell line RAW 264.7 leukaemic monocytes	membrane lysate	ABE-NEM	Merrick *et al*. *MCP*. 2011.	Supp. S10 Table
	dendritic cell line DC2.4	total cell lysate	Bioorthogonal labeling (stearic: 17-ODYA)	Yount *et al*. *Nat Chem Bio*. 2010.	Supp. S1 Table, Supp. S2 Table
Rat					
	cultured embryonic neuronal cells and whole brain	total cell lysate and brain synaptosomal fraction	ABE-NEM	Kang *et al*. *Nature*. 2008.	Supp. S3 Table, Supp. S4 Table, Supp. S5 Table

Supp. = Supplemental

## Results

### Generation of a curated mammalian palmitoylome

15 mammalian proteomic studies (Table 1) were compiled into a single compendium, in which palmitoylated proteins were identified in one of three species (human, mouse, and rat; S1 Table). The consolidated mammalian palmitoylation compendium, or palmitoylome, comprises 1,838 genes (S1 Table). Strikingly, this revealed that nearly 10% of the genes in the genome encode a proteoform that is palmitoylated in human, mouse, or rat, which is much greater than previously predicted or revealed by any individual palmitoyl proteomics study [14,27,32,33,35–45].

**Fig 2:**
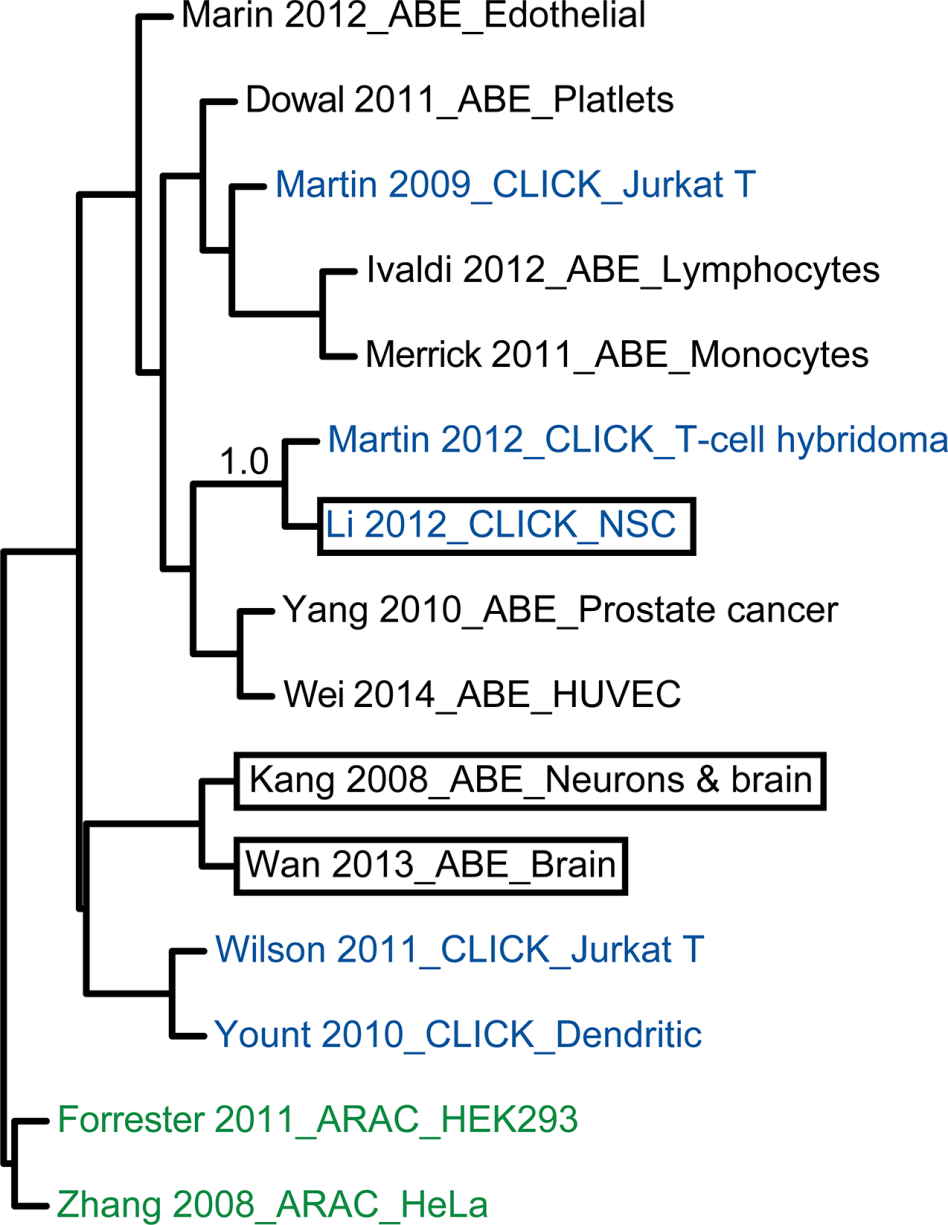
Hierarchical clustering of 15 published palmitoylomes. The gene list of each palmitoyl proteome were subjected to hierarchical clustering using R. Studies that used ABE, bioorthogonal labeling (CLICK) or Acyl-RAC (ARAC) assays are in black, blue, and green font, respectively. Studies that used neuronal sample sources are outlined with black boxes. Bootstrap values are only shown for significant clusters.

In order to determine if there was any bias towards a particular method or biological sample used in the studies included in the compendium, a hierarchical clustering of the 15 palmitoylomes (Fig 2A) was performed using the pvclust package in the statistical software program R [46]. This revealed that there was no apparent clustering based on biological sample or method used, suggesting no technical or biological biases in the data. Indeed, the only statistically significant cluster by resampling involved studies from Martin *et al*. 2012 and Li *et al*. 2012 (bootstrap value = 1.0), which is not surprising as these proteomics experiments were published in the same year from the same laboratory [41,42].

### Enrichment analysis

**Fig 3:**
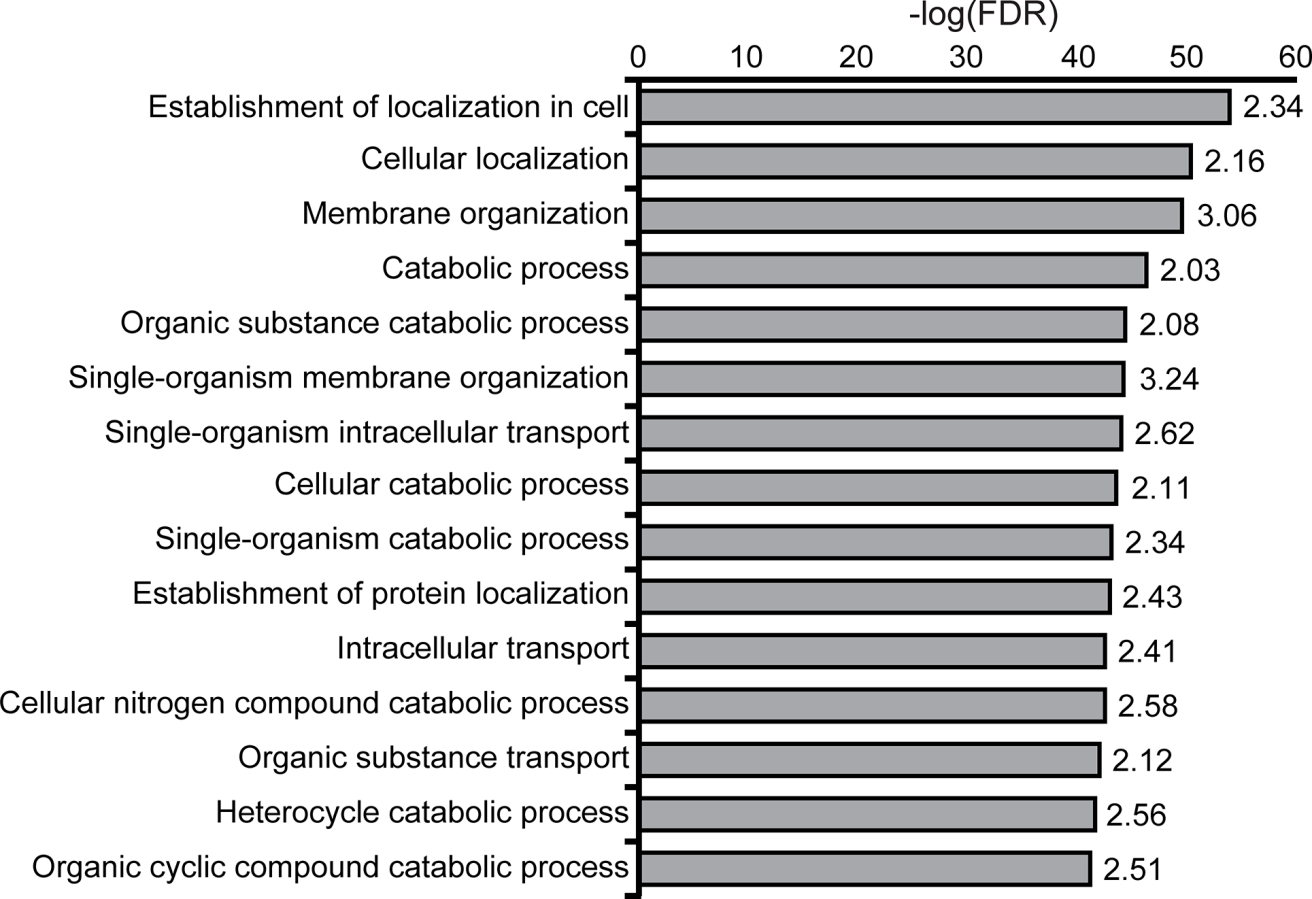
Gene Ontology biological process enrichments among the curated palmitoylome. The top 15 GO biological process enrichments that obtained a FDR<0.001 and a FE≥2 plotted by-log (FDR). The FE is displayed to the right of each bar.

We then investigated the statistical significance of enrichments of GO biological processes [47], metabolic and process networks, pathway maps, and disease-associations in the mammalian palmitoylome (S2–S10 Tables). Protein annotations were considered significantly enriched at a false discovery rate (FDR; multiple hypothesis testing corrected *p*-value) below 0.001 and a fold-enrichment (FE; the ratio of the proportion of palmitoylated proteins with a given annotation over the proportion of proteins with the annotation in the background dataset) greater or equal to 2. Not surprisingly, the top 15 enriched GO biological process annotations were primarily related to protein localization and trafficking (Fig 3 and S10 Table). Enrichment in localization confirms the validity of this approach. Palmitoylation also appeared to be enriched with proteins involved in cell metabolism, which is surprising, as only a small number of proteins involved in metabolism have been shown to be palmitoylated. Interestingly, 36% (662) of the proteins in the compendium are annotated in UniProt as transmembrane proteins whereas only 6% are annotated as peripheral membrane proteins [48]. This suggests that for a large portion of proteins, palmitoylation may play an alternate role other than simply targeting to membranes, such as regulating trafficking, or modulating protein confirmation, protein-protein interactions, or function.

**Fig 4:**
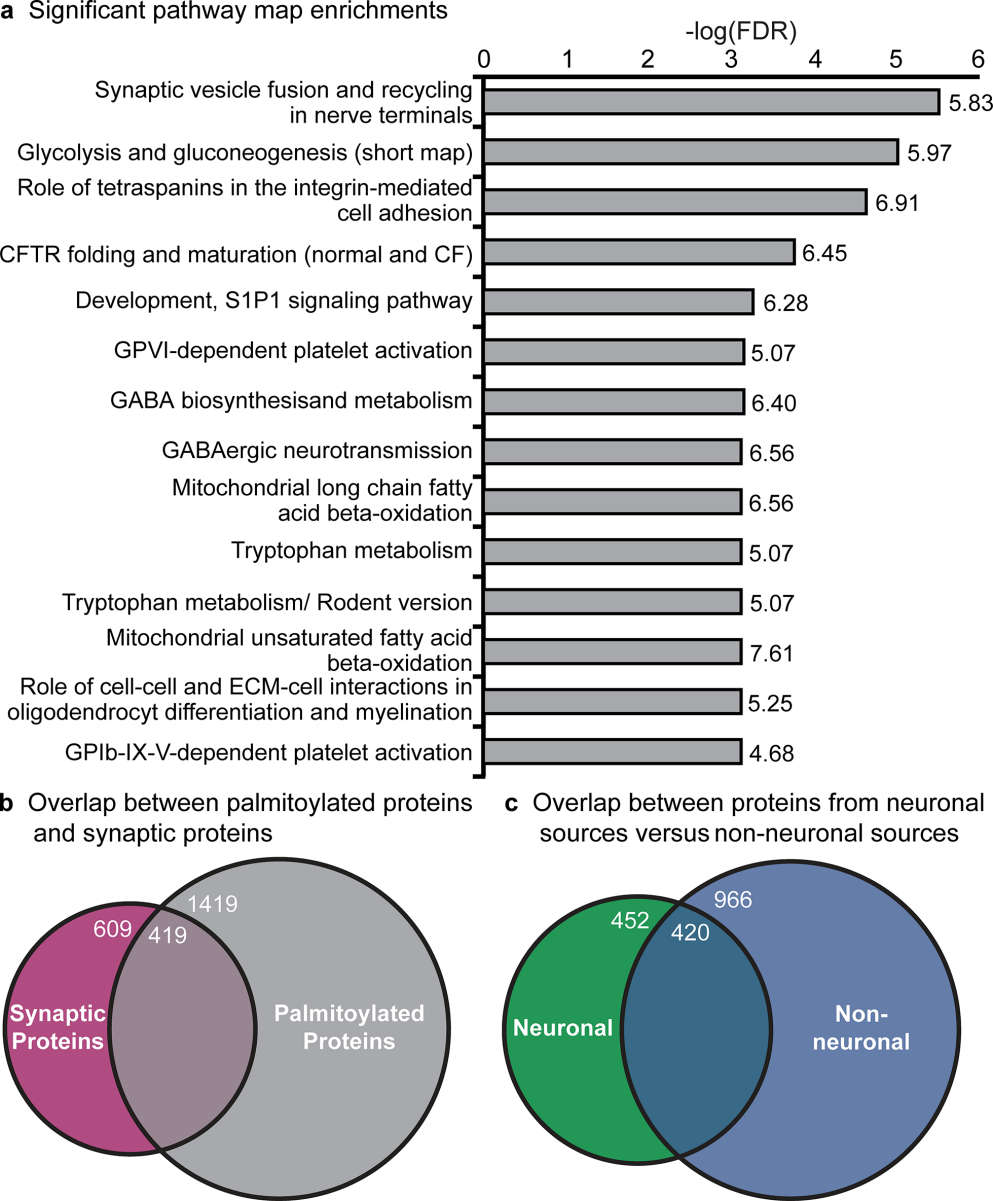
Pathway map enrichments of the curated palmitoylome and enrichment of synaptic proteins for palmitoylated proteins. (**a**) The 14 pathway map enrichments that obtained a FDR < 0.001 and FE ≥ 2 plotted by-log (FDR). The FE is displayed to the right of each bar. Venn diagram of genes in the compendium and the synaptic gene list from SynSysNet is shown in (**b**). The overlap between proteins from neuronal sources versus non-neuronal sources is shown in a Venn diagram in (**c**).

The enrichment analyses also revealed a potential role for palmitoylation in the nervous system, particularly at the synapse. The top MetaCore pathway map annotation was “synaptic vesicle fusion and recycling in nerve terminals” (FDR = 3.11x10^-6^ and FE = 5.83, S8 Table and Fig 4A) suggesting an important role for palmitoylation in neurophysiological processes at the synapse. To investigate further whether palmitoylation is indeed enriched at the synapse, the palmitoylome was compared to the SynSysNet list of 1,028 manually annotated list of genes encoding a synaptic protein [49]. There was a highly significant enrichment of palmitoylated genes in the synaptic gene list versus background (p-value (*p*) = 2.22x10^-16^; 95% CI = 5.22–6.85 fold; Fig 4B), with 419 of the 1,028 (41%; S11 Table) synaptic genes found in the palmitoylation compendium. Overall, this suggests that palmitoylation may play an important role at the synapse and that dysregulation of palmitoylation at the synapse may have detrimental effects.

To confirm that the enrichment of palmitoylated proteins in the synaptic gene list and of synaptic proteins in the palmitoylome is not due to a large portion of the compendium being from a neuronal source, the overlap between those proteins identified from neuronal sources was compared to those identified in non-neuronal sources (Fig 4B). More than 75% (1,386) of the proteins in the compendium were identified in a non-neuronal source with 52% being identified only in a non-neuronal source (966). Only 25% (452) of the genes in the compendium were identified only in a neuronal study.

**Fig 5:**
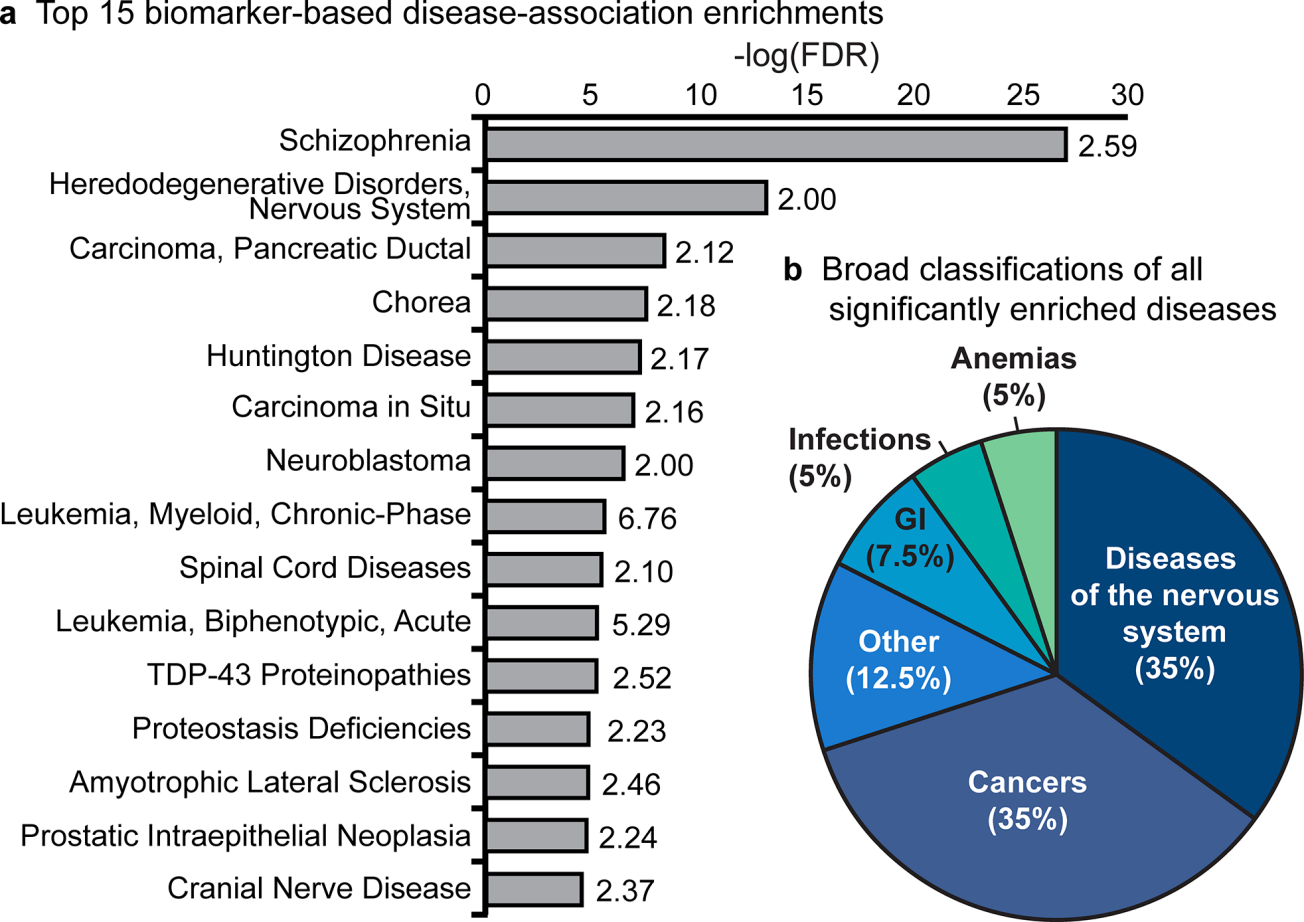
Biomarker-based disease-association enrichments of the curated palmitoylome. (**a**) The top 15 biomarker-based disease-association enrichments that obtained a FDR < 0.001 and a FE ≥ 2 plotted by–log (FDR). The FE is displayed to the right of each bar. All of the 40 statistically significant biomarker-based disease-association enrichments of the compendium were broadly classified and are displayed in (**b**). GI = Gastrointestinal. Other = those diseases that do not fit into any other category.

Finally, the enrichment analysis revealed that a large number of MetaCore biomarker-based disease annotations were significantly associated with nervous system diseases, such as “Schizophrenia” (FDR = 6.39x10^-28^ and FE = 2.61), “Huntington disease” (FDR = 6.09x10^-8^ and FE = 2.46), and “Amyotrophic Lateral Sclerosis” (FDR = 9.59x10^-7^ and FE = 2.57). Cancer annotations, such as “Pancreatic ductal carcinoma” (FDR = 4.37x10^-9^ and FE = 2.12) and “Neuroblastoma” (FDR = 1.47x10^-8^ and FE = 2.00) were also enriched (Fig 5A). When all significantly associated MetaCore disease annotations were broadly classified as diseases of the nervous system, cancers, infections, anemias, gastrointestinal diseases, or other, 14 of the 40 significantly associated MetaCore disease annotations were disease of the nervous system and 14 were cancers (35% each) (Fig 5B and S7 Table).

### Identification of nervous system disease-causing mutations in known or predicted palmitoylated cysteines

The enrichment analyses revealed a potential role for palmitoylation in the nervous system, particularly at the synapse, and in diseases and disorders of the nervous system. Thus we sought to determine whether there were any disease-causing mutations of known or putatively palmitoylated cysteines in genes that cause diseases and disorders of the nervous system. Of the palmitoylated proteins associated with disease phenotypes, superoxide dismutase 1 (SOD1), commonly mutated in hereditary ALS [50], was detected in two studies (S1 Table) and is known to be palmitoylated at cysteine 6 [27,28]. A dominant missense mutation at cysteine 6 of SOD1 was associated with rapid progression in a family with ALS and resulted in a 75% loss of SOD1 activity [51], suggesting that loss of SOD1 palmitoylation may be detrimental (Table 2).

**Table 2:** Disease-causing mutations of known or putatively palmitoylated cysteins in diseases and disorders of the nervous system.

Gene	Mutation	Disease	Cysteine palmitoylated?
*SOD1*	C6F	ALS	Yes
*NPC1*	C74Y	Neimann-Pick disease Type C	Predicted
	C113R		Not predicted but di-Cys likely
	C117G		Not predicted but di-Cys likely
	C670W		Predicted
	C956Y		Predicted
	C1168Y		Predicted
*LGI1*	C42G	Autosomal dominant lateral temporal lobe epilepsy	Not predicted but in Cys-rich region so likely
	C42R		Not predicted but in Cys-rich region so likely
	C46R		Predicted

"Predicted" or "Not predicted" refers to the medium confidence output from CSS-Palm 3.0 palmitoylation site prediction program.

Niemann-Pick C1 (NPC1) was also found to be palmitoylated in four studies (S1 Table). Mutations in NPC1 cause Niemann-Pick disease type C, in which progressive neurological symptoms, including dementia, dystonia, and ataxia, are hallmarks [52]. Two disease-causing mutations in NPC1 involve substitutions of cysteine residues within a di-cysteine motif [53] (Table 2). Such motifs are often palmitoylated [54]. A number of other disease-causing mutations in NPC1 involve other cysteine residues [53] that are predicted to be palmitoylated (Table 2). Of note, NPC1 has also been implicated in AD [52].

Finally, leucine-rich glioma-inactivated protein 1 (LGI1) was detected as palmitoylated in one study. LGI1 cysteine mutations have been associated with autosomal dominant lateral temporal lobe epilepsy [55]. These cysteines are located in a cysteine-rich region likely to contain palmitoylation sites (Table 2) [54].

## Discussion

Herein we present a compendium of palmitoylated proteins curated from 15 previously published proteomics studies aimed at identifying palmitoylated proteins. This compendium is the first curated list of palmitoylated proteins and thus fills the need for such a resource not only in the palmitoylation field but also for the wider research community that may be interested in the post-translational regulation of a given protein. This resource makes proteomics data accessible to researchers attempting to determine if their protein of interest may be palmitoylated. The functional enrichment analysis of the palmitoylated proteins in the compendium is particularly important as it reveals that palmitoylation plays a greater role than previously thought in the nervous system, particularly at the synapse, and in diseases and disorders of the nervous system. This is the first time that the enrichment of palmitoylation in the nervous system has been shown using an unbiased approach. In addition, the significant enrichment of palmitoylated proteins in the synaptic proteome demonstrates that the highly dynamic characteristic of palmitoylation is likely an important regulator of the synapse, where rapid signaling at the membrane is required.

Of particular note, the compendium revealed that 1,838 human genes, or approximately 10% of human genes, encode a proteoform that is palmitoylated. This proportion was surprisingly high as the individual proteomics studies suggested that palmitoylation is much less common as approximately 200–500 proteins were identified in any given study [14,27,32,33,35–45]. The high percentage of genes encoding palmitoylated proteins, in light of the reversibility of this post-translational modification, suggests that palmitoylation acts similarly to phosphorylation for regulation of protein function and localization for a large number of proteins. Like phosphorylation and kinases, this may explain why alterations in palmitoylation are implicated in certain diseases where the regulation of palmitoylation is altered resulting in mislocalization of proteins. For example, alterations in the dynamic nature of RAS palmitoylation and its membrane localization have been implicated in cancers and targeting RAS palmitoylation has been suggested as a potential chemotherapeutic approach [56,57].

This list of 1,838 genes is likely an under-ascertainment, as some proteins may not be fully solubilized during cell lysis and many proteins are not amenable to the repeated protein precipitation/purification steps required for these proteomics studies. Proteins that are of low abundance to begin with or that contain no proteotypic peptides, i.e. peptides that are likely to be detected by MS to identify a protein, would also be difficult to ascertain in any one of these proteomics studies. Additionally, proteins whose palmitoylated proteoforms make up only a very small portion of the total protein population may not be easy to detect in these types of studies. For example, Wan *et al*. identified glutathione synthase (*GS*) and carbonic anhydrase II (*CAII*) as palmitoylated by MS and confirmed that they are palmitoylated using low throughput methods but showed that less than 10% of the protein population of each was palmitoylated [40]. These two proteins were only identified in this one study. Also, some proteins may have an isoform that is expressed in one specific tissue or cell type and not in any other, such as CDC42, which is palmitoylated in the brain and prenylated in other tissues [45]. The reversible nature of palmitoylation may also make it difficult to detect some proteins in one individual study. The power of this meta-analysis comes from the curation of data from many sources into a single list.

The three methods used in these studies to detect palmitoylation (Fig 1) have their various strengths and weaknesses. The bioorthogonal labeling methods are very sensitive but only detect those proteins that are palmitoylated during the limited metabolic labeling period. Thus they detect palmitoylation of proteins that are dynamically palmitoylated or are newly synthesized and palmitoylated during the metabolic labeling time but they do not detect palmitoylation of proteins that are stably palmitoylated and have long half-lives. False positives can arise from the incorporation of the lipid analogue into other lipid modifications other than S-acylation, such as N-palmitoylation, O-palmitoylation, and N-myristoylation, particularly following β-oxidation of the lipid analogue with longer labeling periods. In contrast, the ABE and Acyl-RAC assays detect proteins that are stably palmitoylated and have long half-lives, as they assay the entire population of palmitoylated proteins at a given time. However, the ABE and Acyl-RAC assays are more prone to false positives as hydrolysis of the thioester bond of other cysteine modifications, such as nitrosylation and glutathionylation, or by reduction of disulphide bonds can lead to labeling and false detection.

It is due to these above-mentioned caveats in palmitoylation proteomics studies that the compendium presented here should be used as a starting-off resource for further studies to determine if a protein of interest that appears in the compendium is indeed palmitoylated. The palmitoylation status of any protein in the compendium should be confirmed using multiple low-throughput methods with the appropriate controls. However, proteins identified in more than one study, particularly those identified using more than one method are more likely to truly be palmitoylated. Also, proteins identified in bioorthogonal labeling assays that used hydroxylamine treatment and non-clickable palmitate negative controls (Martin *et al* 2009, Martin *et al* 2012, and Li *et al* 2012) are also more likely to be palmitoylated [33,41,42]. These studies involve the identification of a protein in two different experiments; one using a non-clickable palmitate and the other using hydroxylamine treatment as negative controls and thus are annotated in a separate “methodology” column in the compendium titled “Bioorthogonal labeling (stearic: 17-ODYA) + HAM”. The advantage to using hydroxylamine as a negative control is that it does not eliminate N- and O-acylation modifications thus revealing these types of false positives. However, those proteins that were only identified in one study are still likely to have a proteoform that is palmitoylated. Indeed, a number of proteins that were only identified in a single proteomics study, including DHHC17 [58], DHHC12 [59], PKC epsilon [60], thioredoxin [61], mitochondrial HMG-CoA synthase [4], and β4-integrin [62] have been previously confirmed to be palmitoylated in low-throughput studies.

In the Wilson *et al*. study, various lipid analogues were used, including myristate, palmitate, and stearate [38]. All of the data for this study were included in the compendium as some proteins may be preferentially S-acylated in a fatty acid length dependent manner. Indeed, the PAT DHHC3's activity has been shown to greatly reduce with acyl-CoAs with chains longer than 16-carbons [59]. Of note, only four proteins (FMNL, CHP1, HPCL1, CANB1) were detected using myristate analogues that were not detected using palmitate or stearate or detected in another study. This suggests that most of the proteins detected with myristate in Wilson *et al* are either S-acylated and not myristoylated or are dually acylated proteins that are N-terminally myristoylated and S-acylated elsewhere. This is not surprising, since myristate requires a secondary membrane-binding signal, which typically consists of palmitoylation [63]. In addition, the two N-myristoyltransferases (NMTs) that are responsible for catalyzing N-myristoylation of proteins are highly specific for myristate and do not tolerate longer fatty acids well [63]. Therefore, proteins that were detected using myristate and longer fatty acids are likely to be S-acylated. Consequently, all the proteins detected by Wilson *et al* were included in the compendium. This includes the four proteins not detected in other studies as they may have alternative proteoforms that may be S-acylated. This allows for a more complete and agnostic list that is easy to access and interpret.

Previously, based on the identification of a few palmitoylated proteins, palmitoylation was predicted to be important for synaptic signaling in neurons [64]. Now, for the first time, we demonstrate using an unbiased conservative statistical approach that palmitoylation plays a broad role in synaptic signaling and, consequently, in many diseases and disorders of the nervous system. The fact that the compendium is enriched for synaptic signaling pathways and diseases and disorders of the nervous system was surprising since only three of the 15 proteomics studies included the use of neuronal cells or tissues and 52% of the genes in the compendium were identified only in non-neuronal studies. Despite this, synaptic proteins are significantly enriched for palmitoylated proteins (41%). This significant enrichment of palmitoylated proteins in the synaptic proteome demonstrates that palmitoylation may be an important regulator at the synapse. Alterations of palmitoylation may have detrimental effects specifically at the synapse and this may explain why palmitoylation is enriched for so many diseases of the nervous system. Indeed, we identified a number of cysteine mutations in putative or known palmitoylation sites in a number of diseases and disorders of the nervous system (Table 2), which provide a few examples where loss of palmitoylation of a residue of a protein in humans may lead to disease.

The compendium gene list was also enriched for association with cancers (Fig 5B). Palmitoylation has been previously linked to cancer [11–15], but this is the first time this has been shown using a conservative meta-analysis approach instead of low throughput methods. The fact that the top two enriched classes of diseases are diseases and disorders of the nervous system and cancers is intriguing as they can be considered as two ends of a pendulum of cell growth and death. Neurodegenerative diseases, in particular, involve cell death, whereas cancers involve over-proliferation of cells. In fact, patients with neurodegenerative diseases, particularly HD, AD and Parkinson disease, have a lower incidence of cancers and those who have had cancer have a lower incidence of AD and Parkinson disease [65–67]. The association of palmitoylation with both nervous system diseases and cancers suggests that aberrant palmitoylation may lead to cell death or uncontrolled cell growth depending on the proteins involved. For example, loss of activity of the PAT DHHC17 (also known as Huntingtin interacting protein 14 [HIP14]) has been implicated in HD [58,68] whereas overexpression of DHHC17 may lead to cancer [69]. In addition, the inhibition of palmitoylation of oncogenic proteins such as RAS has been suggested as an avenue for development of chemotherapeutic drugs [56,57].

An interesting role of palmitoylation that came out of the functional enrichment analysis performed here was the association with cell metabolic processes. The role of palmitoylation in cell metabolism may provide a connection between these two types of diseases as metabolic disturbances play a large role in both cancers and diseases and disorders of the nervous system [70,71]. In fact, increased risk of cancer has been linked to both dietary fat intake and increased intracellular levels of palmitate synthesized *de novo* by fatty acid synthase [72,73]. Consequently, a high fat diet or increases in cellular palmitate may alter palmitoylation or lead to increased palmitoylation of many signaling cascades, like ongenic Ras, and potentially promote the cancerous state. In addition, as S-acylation primarily uses palmitate because it is the most abundant lipid in the cell [1,2], it is worth considering that a change in lipid bioavailability due to diet could alter the types of lipids used for S-acylation. This could dramatically affect protein interactions with membranes. Finally, as it has been shown that metabolic proteins may be inhibited by palmitoylation at their actives sites [4], it is possible many proteins may be regulated by palmitate availability. In contrast, loss of palmitoylation by dietary uptake or metabolism or dysregulated pathways in neurodegeneration may lead to cell death. It would be very interesting to know if simply treating with fatty acids could ameliorate some palmitoylation defects in neurodegenerative diseases. Indeed, recent evidence has shown that dietary lipids have a beneficial effect in the treatment of Schizophrenia [74], which was shown to be enriched in our study.

Our meta-analysis suggests that aberrant palmitoylation plays a role in many nervous system diseases and it provides a myriad of putative targets for the treatment of diseases of the nervous system including ALS, Schizophrenia, and HD.

## Materials and Methods

### Published palmitoylated protein data collection and processing

To date, 16 palmitoyl proteomics studies have been performed with three different species: human, mouse, and rat. The data from all but one of these palmitoyl proteomics studies were included. Ren *et al*. was excluded since multiple errors were detected regarding the protein identifiers reported in the supplemental data [75]. With the goal of building a unique consolidated list of palmitoylated proteins, the different identifiers, database accession numbers, and names of the palmitoylated proteins in each dataset of these 15 studies were extracted. The human palmitoylation studies of Dowal *et al*. [37], Forrester *et al*. [32], and Wei *et al*. [35] reported UniProt entry names; Martin *et al*. [33] reported Ensembl Gene IDs; while those of Ivaldi *et al*. [36], Marin *et al*. [27], Wilson *et al*. [38], and Yang *et al*. [39] reported gene names and descriptions. The dataset from Zhang *et al*. [14] reported NCBI RefSeq Accession Numbers [76]. In the mouse studies, Li *et al*. [42], Martin *et al*. [41], Wan *et al*. [40], and Yount *et al*. [77] reported gene names and descriptions of palmitoylated proteins, while Merrick *et al*. [43] reported NCBI protein GI numbers and descriptions. Finally, the rat study of Kang *et al*. [45] reported gene names and descriptions of palmitoylated proteins. “SUSD3_HUMAN” was manually removed from the dataset of Dowal *et al*., as it was labeled as disproved. Also, rows 182–183 from S1A Table and rows 188–198 from S2A Table from Wilson *et al*. were excluded as recommended in that paper, since the signal from these proteins was enriched in the negative controls [38].

In order to consolidate the different lists of palmitoylated proteins described above, the UniProt databases Swiss-Prot and TrEMBL (release-2014_01) [48] and the associated entry name-mappings for human, mouse, and rat were downloaded on February 19, 2014. The UniProt databases were processed to extract the entry name, gene name, full name, Entrez Gene ID, and available synonyms of each protein. Using their respective means of identification, palmitoylated proteins from all studies were matched with the corresponding entry names in the UniProt database. If a protein was matched to a database entry in both TrEMBL and Swiss-Prot, the one from the Swiss-Prot database was given priority, since Swiss-Prot is manually annotated and reviewed and therefore of higher quality. On average, 97.67% of the proteins of a study were matched to the UniProt database entry names, with the minimum being 90.1% for the study from Merrick *et al*. Hence, this compendium of palmitoylated proteins was built from the studies mentioned above in human, mouse, and rat by reporting their gene names and corresponding UniProt entry names and Entrez Gene IDs [78]. The methods of detection of palmitoylated proteins typically do not always allow the differentiation between different protein isoforms, but do permit mapping to a single gene. Therefore gene identifiers were used to curate the compendium. Palmitoylated proteins in the consolidated list that were not reported in all three species had their homologs inferred from the UniProt database based on their UniProt entry names and gene names. The resulting complete consolidated compendium of palmitoylated proteins from the published proteomics studies is reported in S1 Table. When a protein was reported in a given study it is annotated in the compendium under the appropriate study column with a “1”. When a protein was identified in a given study using a particular type of assay it is also annotated in the compendium under the appropriate method column with a “1”. The total number of studies each protein was identified in was annotated under the “Number of Studies Observed in” column. Importantly, these annotations were made on a per study basis not based on the number of times each study identified a given protein.

### Hierarchical clustering

Hierarchical clustering of the consolidated study list was performed using a binary array in which each study was represented by a vector of length equal to the total number of palmitoylated genes. A value of 1 was entered in the vector if a palmitoylated protein was observed in the corresponding study and 0 if it was not. The complete array of study vectors were clustered and plotted with the pvclust package in R [46], using average linkage and binary distance. Bootstrap values were calculated from 5000 samplings.

### Functional enrichment analysis

The MetaCore software package version 6.19 build 65960 (Thomson Reuters) was used to assess the statistical significance of the enrichments of disease-associations, pathway maps, process networks, Gene Ontology (GO) biological processes, and metabolic networks [47] in our compendium of human palmitoylated proteins (MetaCore output files are provided as S2 Table, S3 Table, S4 Table, S5 Table, and S6 Table, respectively). Since the palmitoylated proteins in our dataset were identified using proteomics experiments involving MS, the MetaCore enrichment analysis was performed using the union of two recent human proteome MS-based datasets as background [79,80]. The 32 palmitoylated proteins from our consolidated list that were not present in this background dataset were appended to it. Entrez Gene IDs were obtained for all proteins in this background dataset. This resulted in a background dataset of 17,858 Entrez Gene IDs. Using this dataset as background in lieu of the entire set of human genes has the advantage that biases introduced from the identification of palmitoylated proteins through MS will be considered in the MetaCore enrichment analysis.

For the MetaCore enrichment analysis, protein annotations among palmitoylated proteins that obtained a FDR below 0.001 and a FE greater or equal to 2 were deemed statistically significantly enriched (S7–S10 Tables). The FE, a ratio of the proportion of palmitoylated proteins within a given annotation over the proportion of proteins with the annotation in the background dataset, was used to perform a conservative enrichment analysis and avoid the inclusion of very broad and largely uninformative annotations in our results, which may obtain significant FDRs using the MetaCore analysis. The conservative FE threshold of 2 was based on previously used thresholds in the literature [81–83]. Results from the enrichment analysis for disease-associations, pathway maps, process networks, and GO biological processes are reported in S7, S8, S9, and S10 Tables, respectively. Only one metabolic network annotation, “Lipid metabolism, fatty acid beta-oxidation” was significantly enriched (FDR = 1.45x10^-4^ and FE = 6.02) in the complete list of palmitoylated proteins. The 40 significantly enriched disease associations were broadly classified as diseases of the nervous system, cancers, infections, anemias, gastrointestinal diseases, or other (S7 Table and Fig 5B). Some repetitive disease annotations in S7 Table were not reported.

### Synaptic proteome comparison

Entrez gene names from the compendium were compared to the Entrez gene names in the manually annotated and updated SynSysNet list of 1,028 synaptic genes (downloaded on January 13, 2015) [49]. A Fisher’s exact test was used to assess the statistical significance of the enrichment of palmitoylated genes in the synaptic genes dataset (419 in 1,028) to that in the background proteome dataset (1,838 in 17,858) and to compare the converse enrichment of synaptic genes in the compendium (419 in 1,838) to that in the background dataset (996 in 17,858).

### Mining for nervous system disease-causing cysteine mutations

All genes from the compendium that are associated with diseases of the nervous system were extracted using the OMIM morbid map [84]. OMIM entries corresponding to these genes were then obtained and searched for disease-causing cysteine mutations that were annotated in UniProt, reported in the literature as palmitoylated, or predicted to be palmitoylated by CSS-Palm 3.0 [85].

## Supporting Information

S1 Table Compendium of published mammalian palmitylomes. (XLS)

S2 Table MetaCore Enrichment Analysis output: Biomarker-based disease-association enrichments among all palmitoylated proteins. (XLS)

S3 Table MetaCore Enrichment Analysis output: Pathway map enrichments among all palmitoylated proteins. (XLS)

S4 Table MetaCore Enrichment Analysis output: Process network enrichments among all palmitoylated proteins. (XLS)

S5 Table MetaCore Enrichment Analysis output: Gene Ontology biological process enrichments among all palmitoylated proteins. (XLS)

S6 Table MetaCore Enrichment Analysis output: Metabolic Network enrichments among all palmitoylated proteins. (XLS)

S7 Table Biomarker-based disease-association enrichments among all palmitoylated proteins that obtained a FDR < 0.001 and a FE ≥ 2. (XLSX)

S8 Table Pathway map enrichments among all palmitoylated proteins that obtained a FDR < 0.001 and a FE ≥ 2. (XLSX)

S9 Table Process network enrichments among all palmitoylated proteins that obtained a FDR < 0.001 and a FE ≥ 2. (XLSX)

S10 Table Gene Ontology biological process enrichments among all palmitoylated proteins that obtained a FDR < 0.001 and a FE ≥ 2. (XLSX)

S11 Table Palmitoylated proteins from the palmitoylation compendium that are synaptic proteins. (XLSX)
